# Influenza B viruses circulated during last 5 years in Mongolia

**DOI:** 10.1371/journal.pone.0206987

**Published:** 2018-11-15

**Authors:** Naranzul Tsedenbal, Altansukh Tsend-Ayush, Darmaa Badarch, Sarantuya Jav, Nymadawa Pagbajab

**Affiliations:** 1 National Influenza Center, National Center for Communicable Diseases, Ministry of Health, Ulaanbaatar, Mongolia; 2 School of Bio-Medicine, Mongolian National University of Medical Sciences, Ulaanbaatar, Mongolia; 3 Mongolian Academy of Medical Sciences, Ulaanbaatar, Mongolia; Icahn School of Medicine at Mount Sinai, UNITED STATES

## Abstract

Influenza B virus-caused illness has recently been considered as an urgent public health problem due to substantial morbidity, mortality and life-threatening medical complications. In this study, we have reported the main characteristics of influenza B virus in Mongolia, including prevalence, lineages, suitability with vaccine strains and drug susceptibility against the virus. 15768 specimens were tested by qPCR for detecting influenza viruses. From positive specimens for influenza B virus, the clinical isolates were isolated using MDCK cells. Sequencing analysis, hemagglutination inhibition assay and Neuraminidase inhibitor (NAI) drug susceptibility testing were performed for the clinical isolates. Influenza B virus was around in 3.46% of the samples in Mongolia, and B/Victoria clade-1A and B/Yamagata clade-3 lineages were predominant. Importantly, it was confirmed that the lineages corresponded to the vaccine strains. Moreover, drug susceptibility tests revealed that some Mongolian clinical isolates showed reduced susceptibility to antiviral agents. Interestingly, G104R was identified as a novel mutation, which might have a significant role in drug resistance of the virus. These results describe the characteristics of influenza B viruses that have caused respiratory illness in the population of Mongolia between 2013 and 2017.

## Introduction

Seasonal influenza, caused by influenza A and B viruses, has been reported as one of the urgent public health issues worldwide due to annually substantial morbidity and mortality among the world population [1,2]. Among them, influenza B viruses are known to primarily infect the human population and spreads as an acute febrile illness with respiratory symptoms. In addition, the influenza B viruses lead to severe and life-threatening medical complications in human such as bacterial pneumonia, encephalitis, myositis, Reye’s syndrome and sinus infection [3].

The influenza B virus is a RNA virus, included in the virus family *Orthomyxoviridae* [4,5]. Characterization of the influenza B viruses can be determined by their surface antigens such as hemagglutinin (HA) and neuraminidase (NA). On the basis of the antigenic properties of surface glycoprotein HA, the influenza B virus is classified into two lineages such as B/Victoria and B/Yamagata lineages, which can be frequently used to determine the global circulation of the virus among human population [6]. Many studies showed that the spread and predominance of both lineages of the virus are periodically and geographically different in various regions in the world. Both lineages of the influenza B virus were firstly identified in 1988–1989 and were known to co-circulated globally in 1990s, with B/Yamagata lineage viruses being predominant. Between 1991 and 2000, B/Victoria lineage viruses were primarily identified in eastern Asia. Later, the reappearance of influenza B/Victoria lineage viruses was observed as predominant influenza strain in North America and Europe during 2000–2002 and then spread globally [7–9].

Limiting the impact of disease associated with influenza B virus infections remains an important global issue [5]. Currently, the prevention of the illness is mainly accomplished with trivalent influenza vaccine against the influenza viruses such as two different influenza A type strains (A/H1N1, A/H3N2) and one influenza B type strain. Since the influenza season of 2013/2014, a fourth component was added to develop a quadrivalent vaccine as tool against more influenza types, by including two circulating lineages of influenza B virus for wider protection against influenza [10].

Neuraminidase inhibitors (NAIs) such as oseltamivir and zanamivir have been used as main treatment agents against the influenza B virus infection [11]. In recent years, reduced-susceptibility to NAIs has been reported and has attracted more attention to research as the main concern in the treatment of influenza B virus-caused illness worldwide. These resistant viruses can be emerged under drug selection pressure or occurred naturally without drug interventions. Okomo-Adhiambo et al reported that 346 of influenza B viruses were isolated in 2011 and 2 (0.6%) were identified with reduced susceptibility to NAIs [12–14].

In Mongolia, circulation of influenza B virus was primarily described between 2007 and 2012. During the time period, co-circulation of B/Yamagata and B/Victoria lineages were observed between in the season of 2007 and 2008, and B/Victoria lineages predominated between 2010 and 2012. The antigenicity of influenza B virus strains detected during the periods were considered as well matched to that of the vaccine strains. Since 2010, National Influenza Center of Mongolia has been monitoring NAIs resistance of the influenza B virus by chemiluminescence based assay. As a consequence of the monitoring system, one case with H273Y (resistant to oseltamivir and peramivir) mutation in neuraminidase of the influenza B virus was detected, which occurred naturally [15].

In this study, we aimed to report molecular and antigenic characteristics of the influenza B virus isolated in Mongolia from 2013 to 2017. For this purpose, we firstly analyzed HA sequences of influenza B viruses from databases in the National Center for Communicable Diseases. Then, the phylogenetic relationship of those sequences was explored by focusing on the distinct evolutionary patterns between B/Yamagata and B/Victoria lineages. Further, co-circulation of the viruses from both lineages in Mongolia and the compatibility of the vaccine strains with the virus were examined. Finally, mutations that cause reduced inhibition of anti-influenza B virus agents were analyzed.

## Materials and methods

### Drug and reagents

Oseltamivir carboxylate, zanamivir, laninamivir, and peramivir were kindly provided by F. Hoffmann-La Roche Ltd. (Basel, Switzerland), GlaxoSmithKline (Middlesex, UK), Daiichi Sankyo Co., Ltd. (Tokyo, Japan), and Shionogi Co. Ltd. (Osaka, Japan) respectively.

### Sample collection

A total of 15768 nasal and throat swab specimens were collected from patients with influenza-like illness (ILI) at 164 sentinel surveillance units (outpatient and hospital-based), including the First Central Hospital, the Third Central Hospital, the National Center for Maternal and Child Health, the National Cancer Center, National Center for Communicable Diseases and family hospitals in all 21 provinces and 9 districts of Ulaanbaatar, Mongolia during the influenza seasons of 2013–2017. According to surveillance case definition guideline for influenza like-illness from World Health Organization (WHO), influenza like-illness (ILI) is defined as a sudden onset of fever (≥38°C) within the last 10 days with one or more respiratory symptoms (cough or sore throat). Nasal and throat swab specimens were collected during the 3 days after onset of clinical symptoms and transported in virus transport medium within 72 hours at +4°C to virology laboratory, National Influenza center. The specimens were tested by real-time RT-PCR (qPCR) reactions using an AgPhad-ID One-Step qPCR Kit (Ambion) according to standard protocol for qPCR (WHO and US-CDC) to identify influenza A/B virus [16]. Influenza B virus positive samples were incubated in a Madin-Darby canine kidney (MDCK) cell according to protocol developed by CDC [16].

### Madin-Darby canine kidney cell culture

MDCK cells were grown in Dulbecco’s Modified Eagles medium (DMEM) supplemented with 10% fetal bovine serum, 100 U/ml penicillin, and 100 μg/ml streptomycin and 25 mM HEPES buffer (from Gibco). Then 100 μl of each specimen were inoculated into confluent 3-day-old MDCK cell monolayer, rotated flask to ensure virus covers entire the monolayer and allowed inoculum to adsorb for 30 minutes at 37°C. D-MEM containing 2% bovine serum albumin (BSA), penicillin (100 U/ml), streptomycin (100 μg), HEPES buffer (25 mM) and TPCK-trypsin (2μg/ml) were added for virus growth. Culture flasks incubated at 37°C for 5 days and observed daily for cytopathic effect (CPE). At day 5, hemagglutination inhibition test was performed to define the starting material for the next passage.

### Ethics statement

The study was executed using throat swab specimens which were deposited as anonymous. The research ethics board of the Mongolian National University of Medical Sciences (MNUMS) approved the research (Approval number: 2013/d-11).

### Viral RNA extraction and identification

Viral RNAs were extracted using a QIAamp Viral RNA mini kit (QIAGEN, Hilden, Germany) according to the manufacturer’s instruction.

### Hemagglutinin (HA) and neuraminidase (NA) amplification

Viral RNAs were extracted from a 140 μl sample using a QIAamp Viral RNA mini kit (Qiagen, Hilden, Germany) in accordance to the manufacturer’s instructions. The RNA was eluted with 70 μl of elution buffer. Then, cDNAs were generated using a QIAGEN OneStep qPCR Kit (QIAGEN, Hilden, Germany). Amplification of HA and NA genes was conducted by using influenza B specific primers (S1 File) provided by WHO Collaborating Centers. The qPCR conditions were: at 60°C for 1 min, at 42°C for 10 min, at 50°C for 30 min, at 95°C for 15 min, at 94°C for 30 sec, at 52°C for 30 sec, at 72°C for 1 min, (at 94°C for 30 sec, at 52°C for 30 sec, at 72°C for 1 min) x 35 cycles and at 72°C for 7 min.

### Sequence analysis

NA gene sequencing of the viruses was performed according to standard methods with AB BigDye Terminator v3.1 sequencing kit, Applied Biosystems 3130xl Genetic Analyzer using primers which were obtained through Influenza Reagents Resource, Influenza Division, WHO Collaboration Center, CDC, Atlanta, USA. Nucleotide sequences were edited using Geneious Basic 5.5.6 software and were aligned using Molecular Evolutionary Genetics Analysis software MEGA version 6.0. A phylogenetic tree was constructed using the maximum likelihood method along with the MEGA version 6.0 programs.

### Neuraminidase inhibitor (NAI) susceptibility testing

NAI susceptibility testing was completed using a fluorescence-based enzyme-inhibition assay utilizing the substrate 2’-(4-methylumbelliferyl)-α-D-*N*-acetylneuraminic acid (MUNANA) and CLARIOstar Fluorometer (BMG LABTECH) according to the protocol developed by WHO Influenza Collaboration Center, Melbourne, Australia. The NA inhibitor susceptibility of influenza virus isolates were expressed as the concentration of NA inhibitor needed to reduce NA enzyme activity by 50% (IC_50_). NA inhibition assay data were analyzed using Robosage software comparing test data with the data produced by the reference NA inhibitor sensitive and resistant strains, which were provided by WHO Influenza Collaboration Center, Melbourne, Australia[17].

### Hemagglutination inhibition (HI) test

The hemagglutination assay was done to define the concentration of HA antigen, in which 0.75% of red blood cells from guinea pig were used. Briefly, 50 μL of PBS and 100 μL of virus culture were added into A2 to H12 and A2 to G1 wells, respectively. In a well of H1, 100 μL of PBS was added to control red blood cells. 50 μL from A1 to H1 was taken up and mixed with A2 to H2 wells and done successively for A12 to H12. Subsequently, 50 μL of the red blood cell solution was added and the plates were shaken for 10 seconds. The test result was expressed as HI-positive and HI-negative.

### Data analysis

Statistical analysis and data management were performed using SPSS (Version 11.5; SPSS Inc., Chicago, IL, USA). Descriptive analysis, chi square and *p* value were calculated, and data were expressed as mean ± standard deviation (S.D).

## Results

### Prevalence of influenza B virus

**Fig 1:**
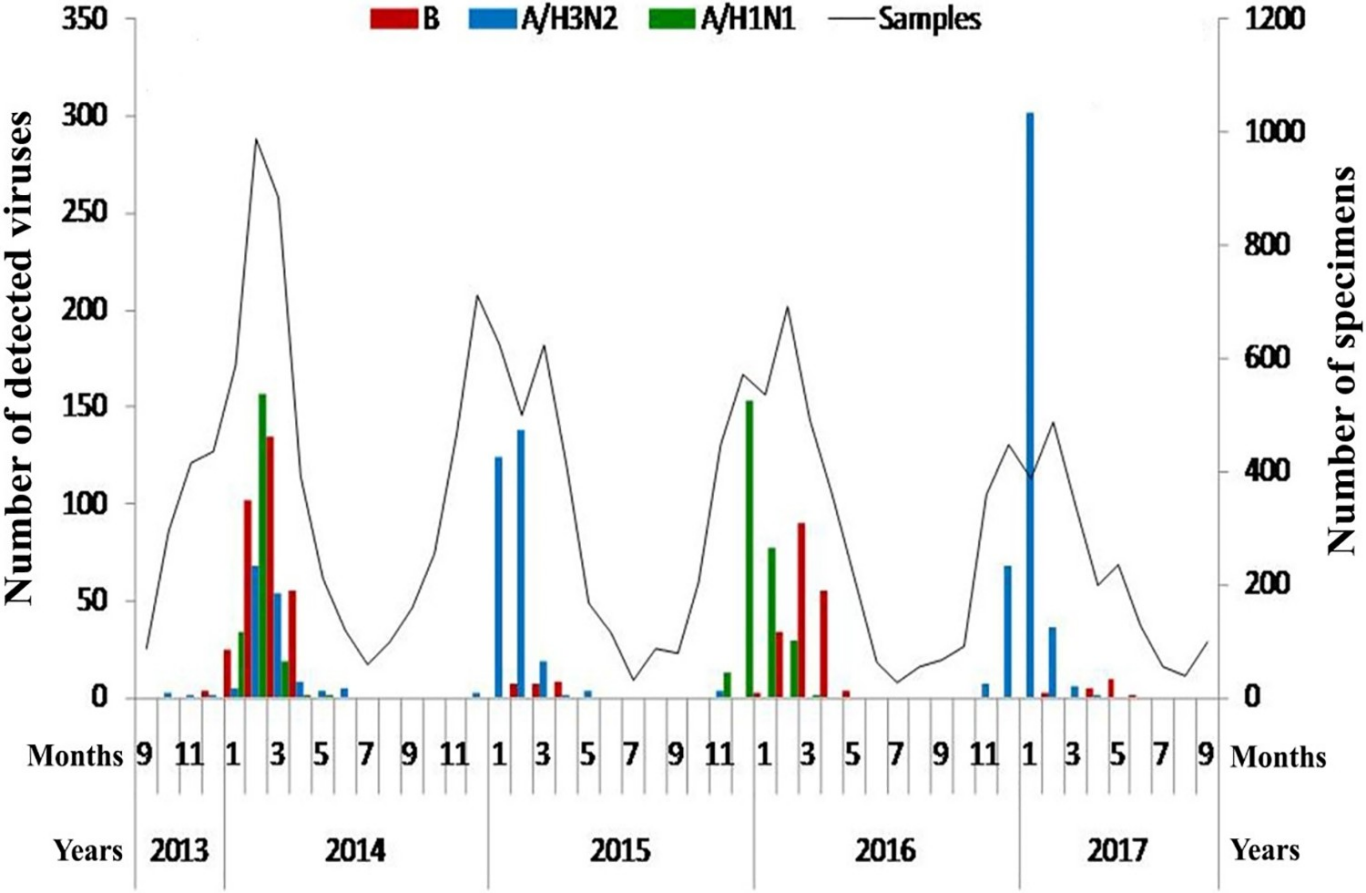
Epidemic curve of influenza virus-positive cases. Monthly distribution of total ILI cases and influenza A and B virus-positive cases in Mongolia from September 2013 to September 2017.

During 2013–2017 in Mongolia, a total 15768 nasal and throat swab specimens were taken from patient with ILI and were investigated for the influenza virus identification. Of these specimens, 546 (3.46%) were confirmed as influenza B virus. 292 (53.5%) and 254 (46.5%) were B/Victoria and B/Yamagata lineages, respectively. The detection rates of influenza B virus were 6.93% (320/4619), 0.58% (24/4117), 4.91% (184/3745) and 0.55% (18/3287) in the influenza season of 2013/2014, 2014/2015, 2015/2016 and 2016/2017, respectively. The 546 patients that confirmed as influenza B virus-positive were aged from newborn to 88 years. The mean and median of ages were 10.24 and 5 years, respectively. Patients aged between newborn to 4 years were most frequently infected by influenza B virus (49.3%), followed by those aged 5–14 years (33%). Of the influenza B virus-positive patients, 239 (43.8%) were male and 307 (56.2%) were female. The infection of influenza B virus was detected as highest rates in March 2014, April 2015 and March 2016 (Fig 1). Between the influenza seasons of 2013 and 2017, B/Victoria and B/Yamagata lineages were revealed at a prevalence of 147(45.9%)/173(54.1%), 0(0%)/22(100%), 127(69%)/57(31%) and 18(100%)/0(0%), respectively (S1 and S2 Tables). During the influenza seasons of 2014/2015, 2015/2016 and 2016/2017, statistically significant differences between the prevalence of B/Victoria and B/Yamagata lineages were observed (*p<0*.*001*). There was no significant difference between the prevalence of B/Victoria and B/Yamagata lineages during the influenza seasons of 2013/2014 (*p>0*.*05*).

**Fig 2:**
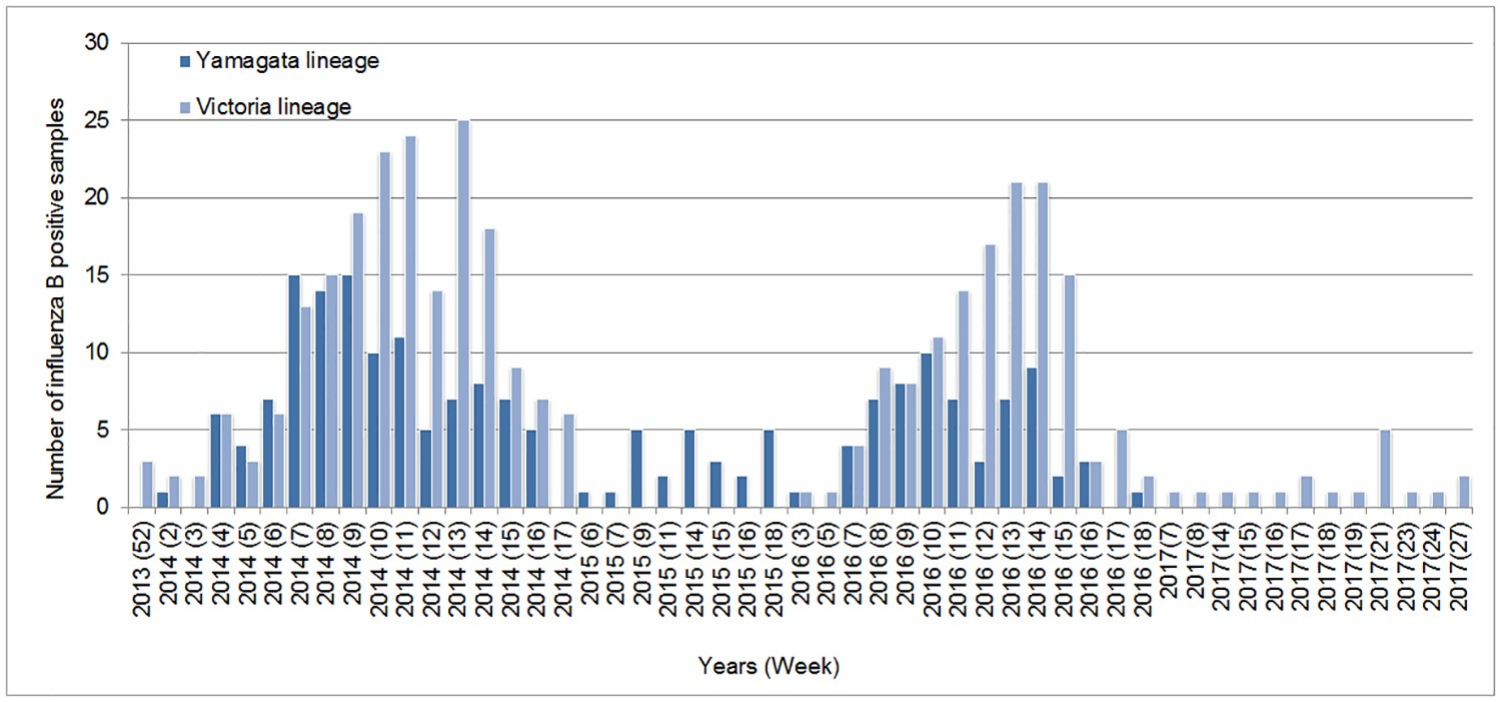
Weekly distribution of influenza B viruses between 2013 and 2017 in Mongolia.

As shown in Fig 2, B/Yamagata and B/Victoria lineages were generally co-circulated during the influenza season of 2013/2014 and 2015/2016, in which B/Victoria lineage was predominantly detected. During the influenza season of 2014/2015, B/Yamagata lineage was predominantly observed, and no B/Victoria lineage was detected. As for the influenza season of 2016/2017, the circulation pattern of influenza B lineages in Mongolia was similarly observed with that in the influenza season of 2014/2015.

### Phylogenetic analysis of HA gene of Mongolian clinical isolates

**Fig 3:**
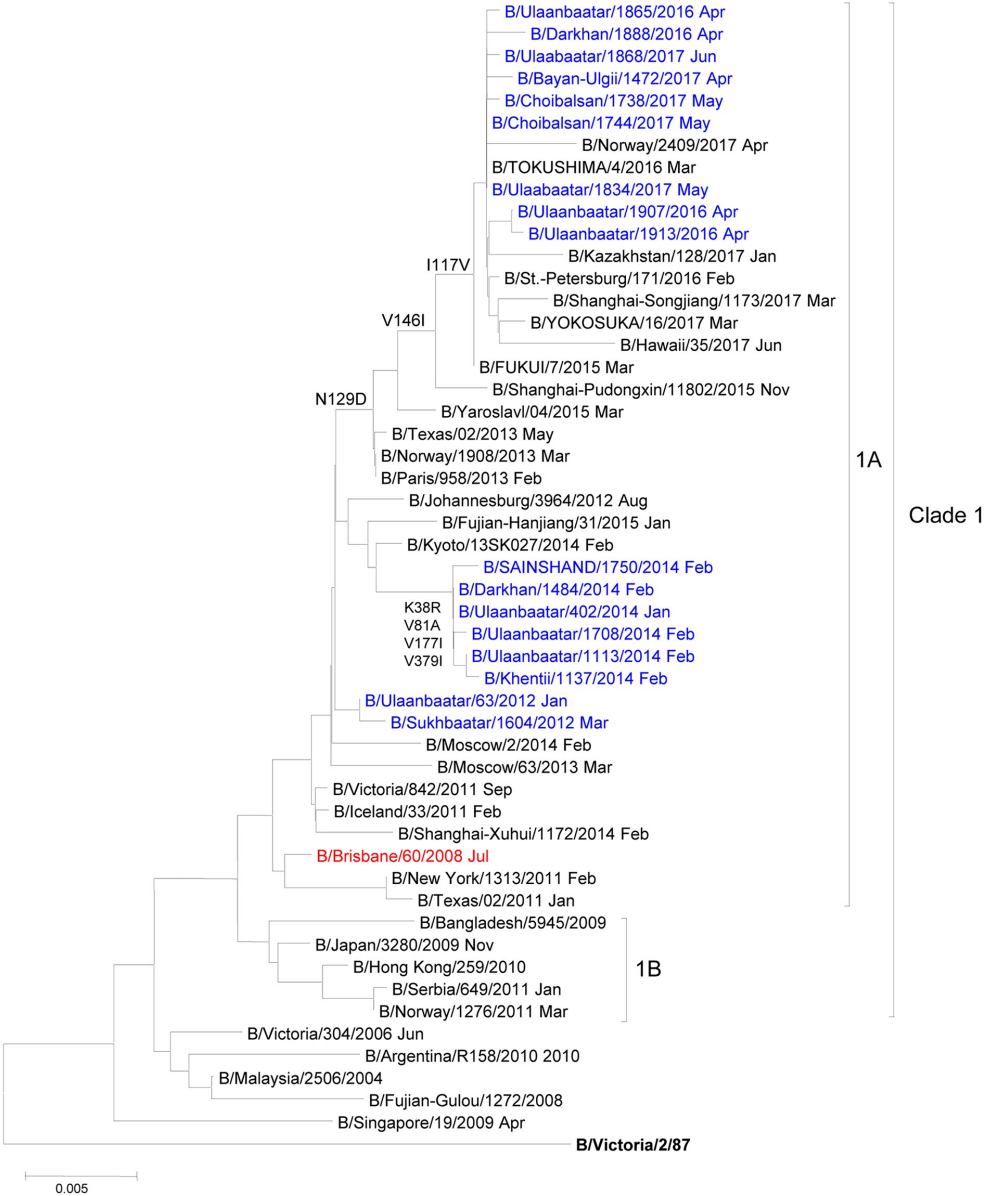
Phylogenetic tree based on HA amino acid sequences of B/Victoria lineage viruses circulated in Mongolia from 2012 to 2017. The phylogenetic tree was created by the neighbor-joining method (1000 bootstrap replicates). Blue is for Mongolian strains, red is for vaccine strains, and black is for reference strains.

**Fig 4:**
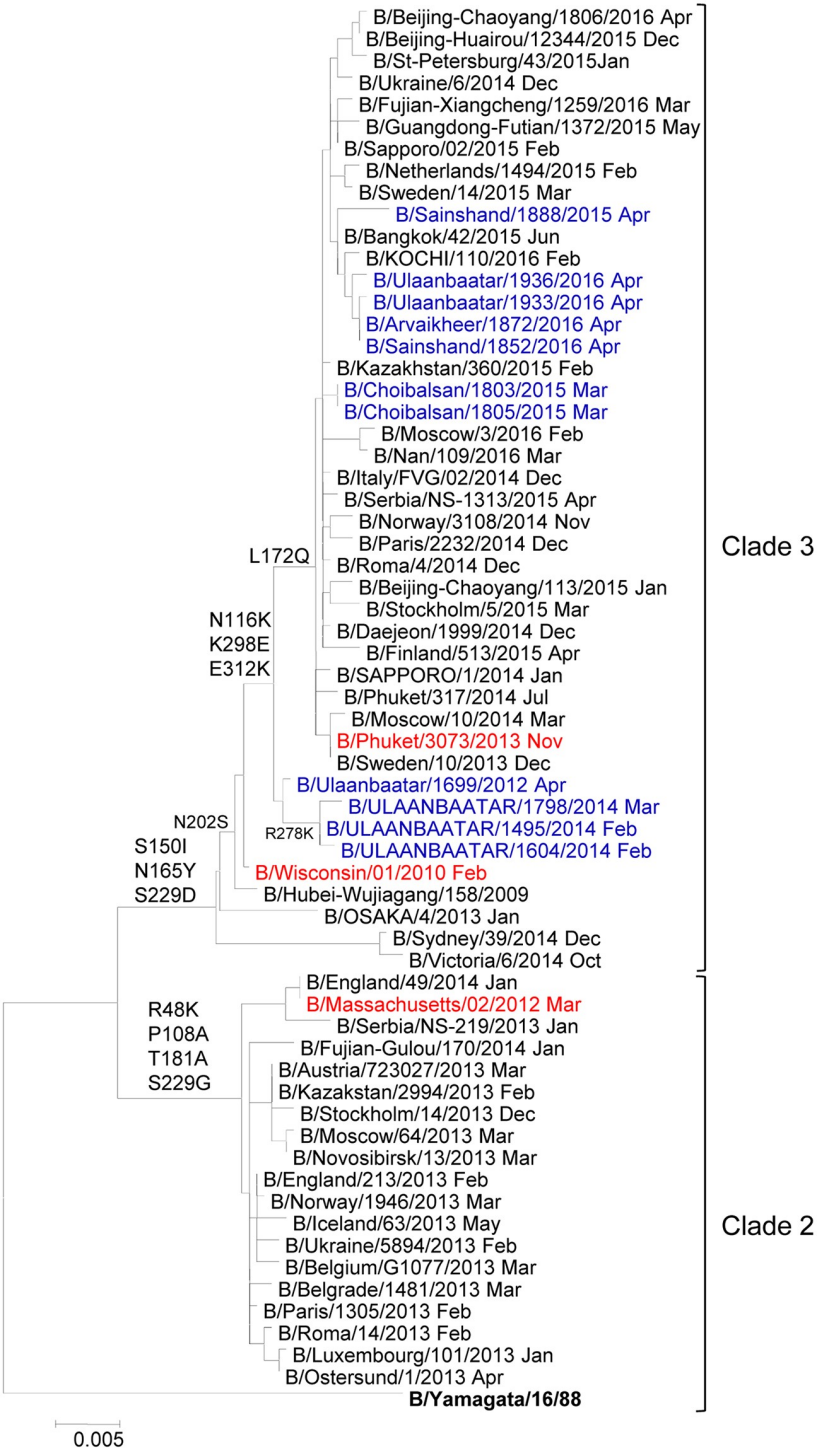
Phylogenetic tree based on HA amino acid sequences of B/Yamagata lineage viruses circulated in Mongolia from 2012 to 2016. The phylogenetic tree was created by the neighbor-joining method (1000 bootstrap replicates). Mongolian strains (blue), vaccine strains (red), reference strains (black).

A total of 25 partial HA sequences were conducted for randomly selected influenza B clinical isolates, isolated from influenza B virus-positive clinical samples in Mongolia. On the basis of HA gene sequence, 15 and 10 clinical isolates were considered to belong to B/Victoria and B/Yamagata lineages, respectively. Phylogenetic analysis showed that all B/Victoria isolates classified into clade-1A, which were corresponded to B/Brisbane/60/2008 as a vaccine strain (Fig 3). On the other hand, all B/Yamagata isolates was classified into clade-3. They were not corresponded to vaccine strain B/Massachusetts/02/2012 but corresponded to vaccine strains B/Wisconsin/01/2010 and B/Phuket/3073/ 2013 (Fig 4).

### Genetic characterization of HA gene of Mongolian clinical isolates

There are four major antigenic epitopes on the membrane distal domain of HA1 subunit of HA which is commonly used for mutation analysis: 120 loop (position 116–137 of HA1), 150 loop (position 141–150), 160 loop (position 162–167), and 190 helix (position 194–202) [18]. Mutation analysis was conducted in the regions for 25 clinical isolates using Geneious software and the results of which were compared to the gene sequences of vaccine strains of the influenza B virus.

In all 15 B/Victoria isolates, amino acid similarity was detected in more than 98% of the isolates as compared to the B/Brisbane/02/2008 as a vaccine strain, whereas all 10 B/Yamagata isolates showed 99% amino acid similarity with B/Phuket/3073/2013 vaccine strain. However, they did not correspond to B/Massachusetts/02/2012, recommended by WHO for the influenza season of 2013/2014.

As shown in Table 1, several amino acid substitutions were found on the HA protein and some mutations were revealed on designated sites of the four key epitopes of HA1. The most represented substitutions were N116K in 120-loop, detected in all 10 B/Yamagata isolates (100%), and I146V in 150-loop was detected in 6 out of 15 (40%) B/Victoria isolates. The substitutions on 160-loop and 190-helix were not found in all isolates.

**Table 1:** Amino acid substitutions of HA protein from influenza B clinical isolates detected in Mongolia during 2013–2016.

Year	Unit	Epitope (Location)	B/Victoria lineage^a^	B/Yamagata lineage^b^
			clade 1 (n = 15)	clade 3 (n = 10)
2013/2014	HA1	120-loop (116–137)	-	N116K (3)
		150-loop (141–150)	I146V (6)	-
		160-loop (162–167)	-	-
		190-helix (194–202)	-	-
2014/2015	HA1	120-loop (116–137)	-	N116K (3)
		150-loop (141–150)	-	-
		160-loop (162–167)	-	-
		190-helix (194–202)	-	-
2015/2016	HA1	120-loop (116–137)	I117V, N129D (4)	N116K (4)
		150-loop (141–150)	-	-
		160-loop (162–167)	-	-
		190-helix (194–202)	-	-
2016/2017	HA1	120-loop (116–137)	I117V, N129D (5)	-
		150-loop (141–150)	-	-
		160-loop (162–167)	-	-
		190-helix (194–202)	-	-

Amino acid substitutions of B/Victoria and B/Yamagata lineages paralleled with the recommended vaccine strains.

(^a^B/Brisbane/60/2008 and ^b^B/Wisconsin/01/2010 are applied for B/Victoria and B/Yamagata, respectively).

### Antigenic characterization of Mongolian clinical isolates

Serological characterization was conducted for B/Victoria isolates using B/Brisbane/60/2008 antisera, and the results compared to the amino acid substitutions in antigenic sites on HA were shown in Table 2. B/Victoria isolates had several amino acid substitutions in 150-loop and in 120-loop, which are known as the main antigenic sites on HA protein. However, no antigenic differences were found between B/Victoria isolates and B/Brisbane/60/2008 vaccine strain by hemagglutination inhibition (HI) tests. B/Victoria isolates in 2014 had amino acid substitutions (I146V) in 150-loop whereas isolates in 2016 had double amino acid substitutions (I117V and N129D) in 120-loop as compared to B/Brisbane/60/2008. The antigenic analysis showed that B/Ulaanbaatar/1913/2016 strain was closer relationship with B/Brisbane/60/2008 than other strains, which might be explained by the amino acid substitution (N197S) in HA protein.

**Table 2:** Antigenic relationship between B/Victoria isolates and B/Brisbane/60/2008 and amino acid substitutions on HA1 gene, refer to B/Brisbane/60/2008 strain.

B/Victoria lineage (Clades)	HI Titer (Antisera)	Amino acid* mutation (B/Brisbane/60/2008)								
		120-loop				150-loop				
		38	81	117	129	146	171	177	197	209
**B/Brisbane/60/2008 (1A)**	**320**	K	V	I	N	I	N	V	N	K
B/Ulaanbaatar/402/2014 (1A)	320	R	A	.	.	V	.	I	.	N
B/ULAANBAATAR/1113/2014 (1A)	320	R	A	.	.	V	.	I	.	N
B/KHENTII/1137/2014 (1A)	320	R	A	.	.	V	.	I	.	N
B/Darkhan/1484/2014 (1A)	320	R	A	.	.	V	.	I	.	N
B/ULAANBAATAR/1708/2014 (1A)	320	R	A	.	.	V	.	I	.	N
B/SAINSHAND/1750/2014 (1A)	320	R	A	.	.	V	.	I	.	N
B/Darkhan/1888/2016 (1A)	320	.	.	V	D	.	.	.	.	.
B/Ulaanbaatar/1865/2016 (1A)	320	.	.	V	D	.	.	.	.	.
B/Ulaanbaatar/1913/2016 (1A)	640	.	.	V	D	.	.	.	S	.
B/Ulaanbaatar/1907/2016 (1A)	320	.	.	V	D	.	.	.	.	.
B/Bayan-Ulgii/1472/2017(1A)	640	.	.	V	D		D	.	.	.
B/Choibalsan/1738/2017(1A)	320	.	.	V	D		.	.	.	.
B/Choibalsan/1834/2017(1A)	320	.	.	V	D		.	.	.	.
B/Ulaanbaatar/1834/2017(1A)	640	.	.	V	D		.	.	.	.
B/Ulaanbaatar/1868/2017(1A)	320	.	.	V	D		.	.	.	.

*(K,Lysine; V, Valine; I, Isoleucine; N, Aspargine; R, Argignine; A, Alanine; D, Aspartic; S, Serine)

Serological characterization was also conducted for B/Yamagata isolates using B/Wisconsin/01/2010, B/Massachusetts/02/2012 and B/Phuket/3073/2013 as reference viruses, and amino acid substitutions in antigenic sites on HA were shown in Table 3. B/Yamagata isolates and B/Phuket/3073/2013 strain had same amino acid substitution (N116K) in 120 loop as, compared to B/Wisconsin/01/2010. All B/Yamagata isolates were closer relationship with B/Phuket/3073/2013, recommended by WHO for use in the influenza season of 2015–2016 and 2016–2017. However, B/Yamagata isolates in 2014 were no relationship with B/Massachusetts/02/2012 recommended by WHO for use in the influenza season of 2013/2014.

**Table 3:** Antigenic relationships between B/Yamagata isolates and B/Wisconsin/01/2010, B/Massachusetts/02/2012 and B/Phuket/3073/2013 and amino acid substitutions on HA1 gene, refer to B/Wisconsin/01/2010 strain.

B/Yamagata lineage (Clades)	HI Titer (Antisera)			Amino-acid* mutation (B/Wisconsin/01/2010)															
				120-loop															
	B/Wisc/01/10	M/Mass/02/12	B/Phuk/3073/13	48	108	116	150	165	172	181	196	202	211	219	229	251	278	298	312
B/Wisconsin/01/2010 (3)	**320**	80	80	R	P	N	I	Y	L	T	N	S	K	V	D	M	R	K	E
B/Massachusetts/02/2012 (2)	80	**640**	40	K	A	.	S	N	.	A	D	N	G	G	G	.	.	.	.
B/Phuket/3037/2013 (3)	320	160	**320**	.	.	K	.	.	.	.	.	.	.	.	.	.	.	E	K
B/Ulaanbaatar/1495/2014 (3)	80	80	320	.	.	K	.	.	.	.	.	.	.	.	.	.	K	E	K
B/Ulaanbaatar/1604/2014 (3)	80	80	320	.	.	K	.	.	.	.	.	.	.	.	.	.	K	E	K
B/Ulaanbaatar/1798/2014 (3)	80	40	320	.	.	K	.	.	.	.	.	.	.	.	.	.	K	E	K
B/Choibalsan/1803/2015 (3)	640	320	640	.	.	K	.	.	Q	.	.	.	.	.	.	.	.	E	K
B/Choibalsan/1805/2015 (3)	320	320	640	.	.	K	.	.	Q	.	.	.	.	.	.	.	.	E	K
B/Sainshand/1888/2015 (3)	320	320	640	.	.	K	.	.	Q	.	.	.	R	.	.	V	.	E	K
B/Sainshand/1852/2016 (3)	160	160	320	.	.	K	.	.	Q	.	.	.	.	.	.	V	.	E	K
B/Ulaanbaatar/1936/2016 (3)	160	160	320	.	.	K	.	.	Q	.	.	.	.	.	.	V	.	E	K
B/Ulaanbaatar/1933/2016 (3)	160	160	320	.	.	K	.	.	Q	.	.	.	.	I	.	V	.	E	K
B/Arvaikheer/1872/2016 (3)	160	160	320	.	.	K	.	.	Q	.	.	.	.	.	.	V	.	E	K

*(R, Argignine; P, Proline; N, Aspargine; I, Isoleucine; Y, Tyrosine; L, Leucine; T, Threonine; S, Serine; K, Lysine; V, Valine; D, Aspartic; M, Methionine; E, Glutamic Acid; A, Alanine; G, Glycine; Q, Glutamine)

### Susceptibility of Mongolian clinical isolates

WHO recommended a category of drug susceptibility definitions by IC50 values in which sensitivity of NAIs was categorized as reduced inhibition and highly reduced inhibition. The reduced inhibition was defined as 5 to 50fold IC 50 values reduction to reference strain and the highly reduced inhibition was defined as more than 50-fold values reduction to reference strain.

During the influenza seasons of 2012–2017, a total of 93 clinical isolates were stored at -70°C for further studies, including 32 isolates from 2013/2014, 3 isolates from 2014/2015, 10 isolates from 2015/2016 and 48 isolates from 2016/2017. Then, 68 influenza B clinical isolates, including 45 isolates from 2013 to 2016 and 23 isolates from the influenza season of 2016/2017, were randomly selected and tested by neuraminidase inhibitors (NAIs) susceptibility testing. A Fluorescence-based assay was used to determine IC50 of oseltamivir, zanamivir, laninamivir and peramivir and all isolates were sensitive to NAIs within the range of 4.53±3.15 nM, 0.76±0.43 nM, 1.12±0.54 nM and 0.47±0.4nM respectively. We have found 5 resistant clinical isolates after several passages through MDCK cells, which were occurred during the influenza season of 2013/2014.

As shown in Table 4, B/Ulaanbaatar/1113/2014 and B/Khentii/1137/2014 were sensitive to oseltamivir, zanamivir, laninamivir whereas they showed reduced susceptibility to peramivir. B/Darkhan/1484/2014 and B/Ulaanbaatar/1495/2014 had reduced susceptibility to oseltamivir, zanamivir and laninamivir whereas highly reduced susceptibility to peramivir. B/Ulaanbaatar/1708/2014 strain had highly reduced susceptibility to all of NAIs tested.

**Table 4:** Result of fluorescence-based NA inhibition assay.

Strains	Oseltamivir		Zanamivir		Laninamivir		Peramivir		Mutation in NA gene
	IC50 (nM)	Fold increase	IC50 (nM)	Fold increase	IC50 (nM)	Fold increase	IC50 (nM)	Fold increase	
B/Ulaanbaatar/1113/2014	9.16	-	0.91	-	1.28	-	11.85	10	I114T
B/Khentii/1137/2014	13.28	-	0.89	-	2.14	-	7.18	10	G140R
B/Darkhan/1484/2014	117.95	15	21.01	10	37.43	16	1525.49	2500	H101N, E105K
B/Ulaanbaatar/1495/2014	58.33	10	7.99	10	61.4	10	3274.91	5000	E105K
B/Ulaanbaatar/1708/2014	3914.6	1000	1451	1000	1532.3	1000	287561.3	>100000	G104R

### Detection of mutations in NA gene of drug resistant Mongolian clinical isolates

NA gene sequences of 5 resistant isolates were browsed in GISAID gene bank with accession numbers: EPI521520; EPI521521; EPI521522; EPI541301; EPI541956. In the sequences of NA for 5 resistant isolates, several novel amino acid substitutions were found in the regions between 101 and 140 amino acids, which belong to enzyme activity site in NA.

**Fig 5:**
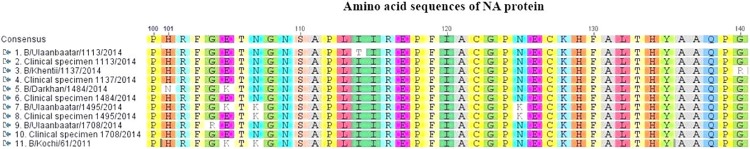
The compared result of drug resistant strains, NA protein sequences with clinical specimens and strain B/Kochi/61/2011.

As shown in Fig 5, I114T mutation was found in B/Ulaanbaatar/1113/2014 strain, which might cause reduced susceptibility to peramivir. G140R mutation was found in B/Khentii/1137/2014 and H101N and E105K mutations were found in B/Darkhan/1484/2014 strain. Besides those, B/Ulaanbaatar/1495/2014 had E105K mutation and B/Ulaanbaatar/1708/2014 had G104R mutation, those also might cause reduced susceptibility to NA inhibitors. These mutations in clinical isolates were not detected in original clinical specimens by sequencing analysis.

## Discussion

Influenza B virus has now been considered as one of the most highly contagious infections for the respiratory system in humans and a main cause of severe respiratory illness and mass hospitalization during the epidemic period [19]. The infection spreads throughout most of Mongolia every winter. The environmental factors such as colder temperature during winter season can contribute the virus survivability by lengthening protective of droplets on viruses [20] thereby increasing the illness caused by influenza B virus in high-risk population. In recent years, the prevention efforts against influenza B have been attracting more attention to limit the virus transmission and reduce illness related to respiratory system. With this regards, WHO recommended vaccination against influenza, including influenza B virus, to prevent the virus-related illness [2,21–23].

In this study, the influenza B virus-caused infection was detected by qPCR and evaluated the prevalence of the virus caused illness in Mongolia between the influenza seasons of 2013 and 2017. During the period, the prevalence of influenza B virus was at 3.46% of all samples collected, indicating that the infection related to influenza B virus contributes a relatively low proportion to seasonal influenza in Mongolia. The patients aged from newborn to 4 years were most repeatedly infected with influenza B virus in Mongolia, which is more likely to be related with child immune system that is not fully developed [24]. In addition, some people are at high risk for seasonal influenza, including children, pregnant women, older people and healthcare workers [18,25].

As regards to the influenza B virus circulation among the population in Mongolia, B/Yamagata and B/Victoria lineages were co-circulated in the influenza season of 2013/2014 and 2015/2016, in which the B/Victoira lineage predominated as compared to B/Yamagata lineage. In the influenza season of 2014/2015, B/Yamagata lineage was exclusively circulated, while the solo-circulation of B/Victoria lineage was observed in the influenza season of 2016/2017. It was considered that the disappearance of two lineages in some season might be related to genetic variability of influenza B viruses [26,27].

WHO and CDC recommended annually anti-influenza vaccination for nearly all people over the ages of six months, especially those at high risk [18,25]. In Mongolia, vaccination against influenza is primarily recommended for high risk groups including pregnant women, elderly, children between six months and five years of age, those with health problem, and those who work in healthcare.

Classification of HA gene based on phylogenetic analysis are more crucial for vaccine selection during influenza season [28,29]. Recently, B/Victoria lineage is classified into clade-1A and clade-1B, and B/Yamagata lineage is classified into clade-2 and clade-3 [28]. In Mongolia, B/Victoria clade-1A was predominantly detected between the influenza seasons of 2013/2014 and 2015/2016, which was well matched to the vaccine strain B/Brisbane/60/2008. B/Yamagata clade-3 was observed in Mongolia during the study period, which was matched to the vaccine strains B/Wisconsin/01/2010 and B/Phuket/3073/2013, not corresponded to the vaccine previous strain B/Massachusetts/02/2012.

The main effort throughout the world to prevent influenza B virus-caused illness is the trivalent vaccine or quadrivalent vaccine, including influenza A and B viruses [21]. The vaccine is more effective when the circulating influenza viruses are well-matched to the vaccine strains recommended by WHO. The difference between antigenic relationship and amino acid substitutions on the HA of Mongolian clinical isolates compared to those of vaccine strains defines the appropriateness of the vaccine strains.

There are several anti-viral drugs such as oseltamivir, zanamivir, laninamivir and peramivir successfully used for the treatment of influenza B virus-caused illness in the world. However, drug resistant influenza B viruses have been recently reported [11,30]. In Mongolia, some influenza B clinical isolates in local regions showed that several resistant isolates to the anti-viral agents, indicating that the treatment against influenza B virus-caused illness might be difficult to prevent. Among Mongolian Influenza B isolates, B/Ulaanbaatar/1708/2014 strain was detected as highly reduced susceptibility to all of NAIs. According to NA sequences, G140R, I114T and E105K mutations were found in Mongolian resistant isolates. Among the mutations, G104R has been occurred during the propagation in MDCK cell culture. Isolate which have G104R mutation showed strong resistant to NAIs as compared to isolates having E105K mutation, suggesting that G104R mutation might play a key role in development of the drug resistance of influenza B virus.

## Conclusions

In Mongolia, influenza B virus has been detected around 3.46% of tested specimens during the study period. Among the influenza B virus, B/Victoria lineage clade-1A and B/Yamagata lineage clade-3 were predominantly observed and mainly caused the illness related to the influenza B virus, which were corresponded to the vaccine strains recommended by WHO. Drug susceptibility tests for Mongolian clinical isolates identified 5 isolates that had reduced susceptibility to antiviral agents. G104R substitution in NA gene of resistant isolate was identified as a novel mutation, responsible for drug resistance. Taking together, these results confirmed the genetic variability and seasonal circulated strains of the influenza B virus isolated in Mongolia and showed that further and continual studies are important to detect the novel genetic variants of epidemiological and clinical significance.

## Supporting information

S1 File Sequencing primers for HA and NA genes. (PDF)

S1 Table Influenza virus-positive cases by year. (PDF)

S2 Table Influenza B virus-positive cases by year. (PDF)

## References

[1] Zhao B, Qin S, Teng Z, Chen J, Yu X, et al (2015) Epidemiological study of influenza B in Shanghai during the 2009–2014 seasons: implications for influenza vaccination strategy. Clinical Microbiology and Infection 21: 694–700. 10.1016/j.cmi.2015.03.009 25882368 10.1016/j.cmi.2015.03.009

[2] Höpping AM, Fonville JM, Russell CA, James S, Smith DJ (2016) Influenza B vaccine lineage selection—an optimized trivalent vaccine. Vaccine 34: 1617–1622. 10.1016/j.vaccine.2016.01.042 26896685 10.1016/j.vaccine.2016.01.042 PMC4793086

[3] Radovanov J, Milošević V, Hrnjaković I, Petrović V, Ristić M, et al (2014) Influenza A and B viruses in the population of Vojvodina, Serbia. Archives of Biological Sciences 66: 43–50.

[4] Bouvier NM, Palese P (2008) The biology of influenza viruses. Vaccine 26: D49–D53. 19230160 10.1016/j.vaccine.2008.07.039 PMC3074182

[5] Paul Glezen W, Schmier JK, Kuehn CM, Ryan KJ, Oxford J (2013) The burden of influenza B: a structured literature review. American journal of public health 103: e43–e51. 10.2105/AJPH.2012.301137 PMC3673513 23327249

[6] Xu C, Chan K-H, Tsang TK, Fang VJ, Fung RO, et al (2015) Comparative epidemiology of Influenza B Yamagata-and Victoria-lineage viruses in households. American journal of epidemiology 182: 705–713. 10.1093/aje/kwv110 26400854 10.1093/aje/kwv110 PMC4715237

[7] Tan Y, Guan W, Lam TT-Y, Pan S, Wu S, et al (2013) Differing epidemiological dynamics of influenza B virus lineages in Guangzhou, southern China, 2009–2010. Journal of virology 87: 12447–12456. 10.1128/JVI.01039-13 24027322 10.1128/JVI.01039-13 PMC3807886

[8] Rota PA, Wallis TR, Harmon MW, Rota JS, Kendal AP, et al (1990) Cocirculation of two distinct evolutionary lineages of influenza type B virus since 1983. Virology 175: 59–68. 2309452 10.1016/0042-6822(90)90186-u

[9] Tewawong N, Suwannakarn K, Prachayangprecha S, Korkong S, Vichiwattana P, et al (2015) Molecular epidemiology and phylogenetic analyses of influenza B virus in Thailand during 2010 to 2014. PloS one 10: e0116302 10.1371/journal.pone.0116302 25602617 10.1371/journal.pone.0116302 PMC4300180

[10] Barr IG, Jelley LL (2012) The coming era of quadrivalent human influenza vaccines. Drugs 72: 2177–2185. 10.2165/11641110-000000000-00000 23110610 10.2165/11641110-000000000-00000

[11] Burnham AJ, Baranovich T, Govorkova EA (2013) Neuraminidase inhibitors for influenza B virus infection: efficacy and resistance. Antiviral research 100: 520–534. 10.1016/j.antiviral.2013.08.023 24013000 10.1016/j.antiviral.2013.08.023 PMC3850058

[12] Sheu TG, Deyde VM, Okomo-Adhiambo M, Garten RJ, Xu X, et al (2008) Surveillance for neuraminidase inhibitor resistance among human influenza A and B viruses circulating worldwide from 2004 to 2008. Antimicrobial agents and chemotherapy 52: 3284–3292. 10.1128/AAC.00555-08 18625765 10.1128/AAC.00555-08 PMC2533500

[13] Okomo-Adhiambo M, Sleeman K, Lysén C, Nguyen HT, Xu X, et al (2013) Neuraminidase inhibitor susceptibility surveillance of influenza viruses circulating worldwide during the 2011 Southern Hemisphere season. Influenza and other respiratory viruses 7: 645–658. 10.1111/irv.12113 23575174 10.1111/irv.12113 PMC5781198

[14] Hatakeyama S, Ozawa M, Kawaoka Y (2011) In vitro selection of influenza B viruses with reduced sensitivity to neuraminidase inhibitors. Clinical Microbiology and Infection 17: 1332–1335. 10.1111/j.1469-0691.2010.03313.x 20636420 10.1111/j.1469-0691.2010.03313.x PMC2980859

[15] Burmaa A, Darmaa B, Naranzul T, Bayasgalan N, Nyamaa G, et al (2012) Influenza surveillance study in Mongolia, 2011/2012 influenza season. Mongolian Journal of Infectious Diseases Research 6: 5–10.

[16] Organization WH (2011) Manual for the laboratory diagnosis and virological surveillance of influenza.

[17] Leang S-K, Hurt AC (2017) Fluorescence-based Neuraminidase Inhibition Assay to Assess the Susceptibility of Influenza Viruses to The Neuraminidase Inhibitor Class of Antivirals. Journal of visualized experiments: JoVE. 10.3791/55570 PMC5564701 28448045

[18] Jorgensen P, Mereckiene J, Cotter S, Johansen K, Tsolova S, et al (2017) How close are countries of the WHO European Region to achieving the goal of vaccinating 75% of key risk groups against influenza? Results from national surveys on seasonal influenza vaccination programmes, 2008/2009 to 2014/2015. Vaccine. 10.1016/j.vaccine.2017.12.019 PMC5777640 29287683

[19] de Barros ENC, Cintra O, Rossetto E, Freitas L, Colindres R (2016) Patterns of influenza B circulation in Brazil and its relevance to seasonal vaccine composition. The Brazilian Journal of Infectious Diseases 20: 81–90. 10.1016/j.bjid.2015.09.009 26626166 10.1016/j.bjid.2015.09.009 PMC7110561

[20] Oong XY, Ng KT, Lam TT-Y, Pang YK, Chan KG, et al (2015) Epidemiological and evolutionary dynamics of influenza B viruses in Malaysia, 2012–2014. PloS one 10: e0136254 10.1371/journal.pone.0136254 26313754 10.1371/journal.pone.0136254 PMC4552379

[21] Pan Y, Deem MW (2016) Prediction of influenza B vaccine effectiveness from sequence data. Vaccine 34: 4610–4617. 10.1016/j.vaccine.2016.07.015 27473305 10.1016/j.vaccine.2016.07.015

[22] Moa AM, Chughtai AA, Muscatello DJ, Turner RM, MacIntyre CR (2016) Immunogenicity and safety of inactivated quadrivalent influenza vaccine in adults: A systematic review and meta-analysis of randomised controlled trials. Vaccine 34: 4092–4102. 10.1016/j.vaccine.2016.06.064 27381642 10.1016/j.vaccine.2016.06.064

[23] Korsun N, Angelova S, Gregory V, Daniels R, Georgieva I, et al (2017) Antigenic and genetic characterization of influenza viruses circulating in Bulgaria during the 2015/2016 season. Infection, Genetics and Evolution 49: 241–250. 10.1016/j.meegid.2017.01.027 28132927 10.1016/j.meegid.2017.01.027 PMC5348111

[24] Pawelec G (2017) Age and immunity: What is “immunosenescence”? Experimental gerontology. 10.1016/j.exger.2017.10.024 29111233

[25] Kunisaki KM, Janoff EN (2009) Influenza in immunosuppressed populations: a review of infection frequency, morbidity, mortality, and vaccine responses. The Lancet infectious diseases 9: 493–504. 10.1016/S1473-3099(09)70175-6 19628174 10.1016/S1473-3099(09)70175-6 PMC2775097

[26] Baker SF, Nogales A, Finch C, Tuffy KM, Domm W, et al (2014) Influenza A and B virus intertypic reassortment through compatible viral packaging signals. Journal of virology 88: 10778–10791. 10.1128/JVI.01440-14 25008914 10.1128/JVI.01440-14 PMC4178878

[27] Dudas G, Bedford T, Lycett S, Rambaut A (2014) Reassortment between influenza B lineages and the emergence of a coadapted PB1–PB2–HA gene complex. Molecular biology and evolution 32: 162–172. 10.1093/molbev/msu287 25323575 10.1093/molbev/msu287 PMC4271528

[28] Mosnier A, Daviaud I, Casalegno J, Ruetsch M, Burugorri C, et al (2017) Influenza B burden during seasonal influenza epidemics in France. Medecine et maladies infectieuses 47: 11–17. 10.1016/j.medmal.2016.11.006 28062245 10.1016/j.medmal.2016.11.006

[29] Arencibia A, Piñón A, Acosta B, Fernandez L, Muné M, et al (2018) Vaccine-mismatched influenza B/Yamagata lineage viruses in Cuba, 2012–2013 season. Infection, Genetics and Evolution 58: 110–114. 10.1016/j.meegid.2017.12.004 29229320 10.1016/j.meegid.2017.12.004

[30] Hurt AC, Besselaar TG, Daniels RS, Ermetal B, Fry A, et al (2016) Global update on the susceptibility of human influenza viruses to neuraminidase inhibitors, 2014–2015. Antiviral research 132: 178–185. 10.1016/j.antiviral.2016.06.001 27265623 10.1016/j.antiviral.2016.06.001 PMC5357725

