# Supplementary material for: Influenza B viruses circulated during last 5 years in Mongolia
Source: PLoS One. 2018 Nov 15;13(11):e0206987. doi: 10.1371/journal.pone.0206987 (PMC6237300; doi:10.1371/journal.pone.0206987)
Supplement: S1 File — (PDF) [file pone.0206987.s001.pdf]

S1 File. Sequencing primers for HA and NA genes

Sequencing primers for HA gene		
Amplification primers for NA gene	B_HA_F1_7	TGTAAAACGACGGCCAGTGCAGAGCATTTTCTAA
	B_HA_R1_538	CAGGAAACAGCTATGACCCCCAAGCCATYTTTGC
	B_HA_F2_226	TGTAAAACGACGGCCAGTAGAGGRAAACTATGYCC
	B_HA_R2_886	CAGGAAACAGCTATGACCCACCACACYTTTTGAGG
	B_HA_F3_453	TGTAAAACGACGGCCAGTGGAACCTCARGRTCTTGCCCTAA
	B_HA_R3_1126	CAGGAAACAGCTATGACCACCAGCAATAGCTCCRAA
	B_HA_F4_738	TGTAAAACGACGGCCAGTCCARAYCAAACAGAAGACGGA
	B_HA_R4_1324	CAGGAAACAGCTATGACCTGGAGTTCATCCATRGC
	B_HA_F5_955	TGTAAAACGACGGCCAGTAAYAAAAGCAAGCCTTACTACAC
	B_HA_R5_1603	CAGGAAACAGCTATGACCTCAAARGTGGGRAGAGAAAA
	B_HA_F6_1264	TGTAAAACGACGGCCAGTGTAAAGAAYCTTCAAAGACTAA
	B_HA_R6_1869	CAGGAAACAGCTATGACCAGTAGTAACAAGAGCAT
Sequencing primers	M13F	TGTAAAACGACGGCCAGT
	M13R	CAGGAAACAGCTATGACC

Sequencing primers for NA gene		
Amplification primers for NA gene	B_NA_F1_1	TGTAAAACGACGGCCAGTAGCAGAAGCAGAGCATCTTC
	B_NA_R1_535	CAGGAAACAGCTATGACCATCATGRCATGCGGACCCGCT
	B_NA_F2_340	TGTAAAACGACGGCCAGTGCACTCCTAATTAGCCC
	B_NA_R2_749	CAGGAAACAGCTATGACCGATAACAATYTCCYCCGATGC
	B_NA_F3_450	TGTAAAACGACGGCCAGTACAGAAACAAGCTGAGRCATCTA
	B_NA_R3_1080	CAGGAAACAGCTATGACCTTCGRGAGTACCAYCTTCCAATYTTGG
	B_NA_F4_676	TGTAAAACGACGGCCAGTAAGAAAGTGCYTGYAATTGCATCG
	B_NA_R4_1318	CAGGAAACAGCTATGACCCCCAATACAGGGGACATC
	B_NA_F5_1026	TGTAAAACGACGGCCAGTGGACACYCCCAGACCARATG
	B_NA_R5_1545	CAGGAAACAGCTATGACCAGTAGTAACAAGAGCAT
Sequencing primers	M13F	TGTAAAACGACGGCCAGT
	M13R	CAGGAAACAGCTATGACC
