# Supplementary material for: Influenza B viruses circulated during last 5 years in Mongolia
Source: PLoS One. 2018 Nov 15;13(11):e0206987. doi: 10.1371/journal.pone.0206987 (PMC6237300; doi:10.1371/journal.pone.0206987)
Supplement: S1 Table — (PDF) [file pone.0206987.s002.pdf]

S1 Table. Influenza virus-positive cases by year

Year	Samples	Positive	A/H1N1	A/H3N2	B
2013/2014	4619	678(36.1%)	211(31.1%)	146(21.5%)	321(47.3%)
2014/2015	4117	310(16.5%)	0(0%)	288(92.9%)	22(7.1%)
2015/2016	3745	454(24.1%)	266(58.6%)	3(0.7%)	185(40.7%)
2016/2017	3287	438(23.3%)	0(0%)	420(95.9%)	18(4.1%)
Total	15768	1880(100%)	477(25.4%)	857(45.6%)	546(29%)
