# Supplementary material for: Influenza B viruses circulated during last 5 years in Mongolia
Source: PLoS One. 2018 Nov 15;13(11):e0206987. doi: 10.1371/journal.pone.0206987 (PMC6237300; doi:10.1371/journal.pone.0206987)
Supplement: S2 Table — (PDF) [file pone.0206987.s003.pdf]

S2 Table. Influenza B virus-positive cases by year

Year	B	Victoria	Yamagata
2013/2014	321(100%)	147(45.8%)	174(54.2%)
2014/2015	22(100%)	0(0%)	22(100%)
2015/2016	185(100%)	127(68.6%)	58(31.3%)
2016/2017	18(100%)	18(100%)	0(0%)
Total	546(100%)	292(53.%)	254(46.5%)
